# PROPER: Performance visualization for optimizing and comparing ranking classifiers in MATLAB

**DOI:** 10.1186/s13029-015-0047-1

**Published:** 2015-12-03

**Authors:** Samad Jahandideh, Fatemeh Sharifi, Lukasz Jaroszewski, Adam Godzik

**Affiliations:** Bioinformatics and Systems Biology Program, Sanford Burnham Prebys Medical Discovery Institute, 10901 N Torrey Pines Rd, La Jolla, CA 92307 USA; Division of Computer Science, School of Informatics and Computing, Indiana University, Bloomington, IN 47405 USA

**Keywords:** Predictive modeling, Scoring classifier, Big data, Structural genomics

## Abstract

**Background:**

One of the recent challenges of computational biology is development of new algorithms, tools and software to facilitate predictive modeling of big data generated by high-throughput technologies in biomedical research.

**Results:**

To meet these demands we developed PROPER - a package for visual evaluation of ranking classifiers for biological big data mining studies in the MATLAB environment.

**Conclusion:**

PROPER is an efficient tool for optimization and comparison of ranking classifiers, providing over 20 different two- and three-dimensional performance curves.

**Electronic supplementary material:**

The online version of this article (doi:10.1186/s13029-015-0047-1) contains supplementary material, which is available to authorized users.

## Background

One of the main challenges of computational biology is developing new algorithms, tools and software to facilitate analysis of Big Data generated by biomedical research. Ranking or scoring predictors play central roles in a very wide range of biological data analysis problems, e.g. protein structure and function annotation, sequence alignment, genome annotation and many others. Most of biological datasets contain extensive noise that complicates predictive modeling. In different applications optimal predictors may differ depending on the purpose of specific study and characteristics of specific datasets. Thus, optimization and comparison of different prediction methods, selection and evaluation of importance of different features, and simple and efficient validation of predictors’ performance is crucial for successful application of machine learning algorithms.

To assist in this task, PROPER provides visual monitoring of optimization and comparison of ranking classifiers. PROPER also allows feature selection and evaluation of features’ importance using l1-regularized logistic regression [1] and Random Forest [2], respectively. At the same time PROPER allows semi-automated optimization of complex methods, such as Artificial Neural Network (ANN). Moreover, output of scoring classifiers currently not implemented in PROPER can be uploaded and used for performance visualization and comparison with available methods.

## Implementation

**Fig. 1:**
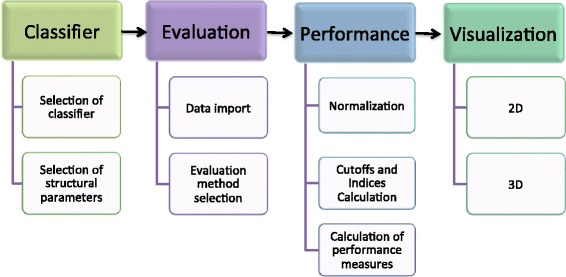
Different steps implemented in PROPER package to visualize and compare performance of classifiers. After selection of a classifier type and evaluation method, PROPER calculates performance measures and visualizes the results as 2D and 3D graphs

PROPER is a flexible classifier evaluation package implemented in MATLAB (http://www.mathworks.com), a statistical language that is widely used in biomedical data analysis. PROPER imports datasets, applies selected classifiers, evaluates them using different methods, calculates performance measures, and visualizes the results (Fig. 1). Several classifiers and evaluation methods are implemented in PROPER’s prediction function, and calculation of performance measures is implemented in performance function. Also, visualization of outputs is implemented in visualization2D function and visualization3D function. All optional parameters of PROPER can be modified by a user when these functions are executed. Graphs plotted using PROPER can be modified and annotated using the MATLAB Plot Editor, which provides user-friendly graphical interface and allows producing high quality figures for publication (for details see PROPER manual (Additional file 1) available at https://sourceforge.net/projects/PROPER-Package).

## Results and discussion

**Fig. 2:**
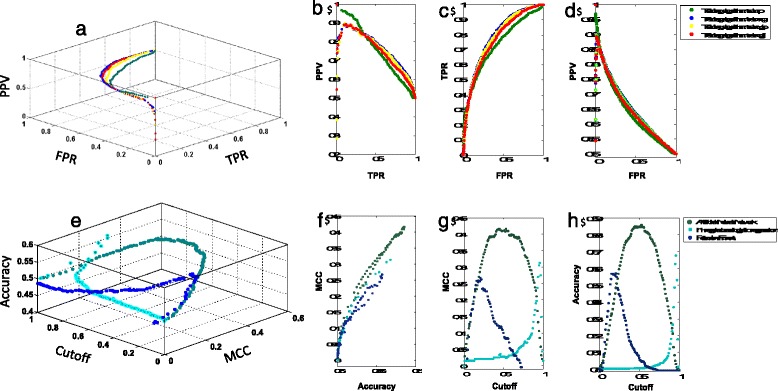
PROPER applied in performance visualization, optimization and comparisons of scoring classifiers on structural genomics data. **a**-**d** optimizing structure of ANN by different training algorithms: **a** an example of three-dimensional performance curve where each facet represents a standard two-dimensional performance curve, e.g. **b** precision-recall curve, **c** ROC curve, and **d** PPV-FPR curve. **e**-**h** comparing performance of three different scoring classifiers: **e** three-dimensional combination of performance curves where each facet represents a two-dimensional performance curve; **f** MCC against Accuracy; **g** MCC against cutoff, and **h** Accuracy against cutoff. According to this result, ANN is a stronger predictor with the highest MCC and accuracy. In this figure, ROC, MCC, TPR, FPR, and PPV represent Receiver Operating Characteristic, Matthews Correlation Coefficient, sensitivity, False Positive Rate, and Positive Predictive Value, respectively

In PROPER the scoring output of ranking classifiers is translated into a binary class decision by applying a spectrum of cutoffs. Usually no specific cutoff can optimally satisfy all possible performance criteria, hence cutoff choice involves a trade-off between different measures. Typically, a trade-off between a pair of measures (e.g. precision versus recall) is visualized as a cutoff-parametrized curve in the plane spanned by the two measures. Many machine learning and statistical learning packages, e.g. Weka [3] and SLEP [1] are available, but none of them offers standardized comprehensive optimizing, comparison, and performance evaluation of biological classifiers. As no cutoff is optimal according to all possible performance criteria, PROPER allows plotting cutoff-parameterized performance curves for any pair of more than 13 predictors’ performance measures and may also plot three-dimensional performance curves by combining three different performance measures in a 3D graph where each facet represents a standard performance curve (Fig. 2).

Calculated performance measures used to comprehensively evaluate the performance of classifiers include:

$$ T= True\  Predictions=\left( True\  Positives+ True\  Negatives\right) $$

$$ F= False\  Predictions=\left( False\  Positives+ False Negatives\right) $$

$$ TPR= Sensitivity= Recall=\kern0.75em \frac{\  True\kern0.5em  Positives}{\left( True\  Positives + False\  Negatives\right)} $$

$$ FNR= False\  Negative\  Rate = \frac{False\  Negative s}{\left( True\  Positives + False\  Negative s\right)} $$

$$ FPR= False\  Positive\  Rate= Fallout = \frac{False\  Positive s}{\left( True\  Negatives + False\  Positive s\right)} $$

$$ TNR= Negative\  Negative\  Rate= Specificity = \frac{\  True\  Negative s}{\left( True\  Negative s + False\  Positives\right)} $$

$$ PPV= Positive\  Predictive\  Value = \frac{\  True\  Positive s}{\left( True\  Positive s + False\  Positive s\right)} $$

$$ NPV= Negative\  Predictive\  Value = \frac{\  True\  Negative s}{\left( True\  Negative s + False\  Negative s\right)} $$

$$ RPP= Rate\  of\  Positive\  Prediction = \frac{\  True\  Positive s + False\  Positive s}{\left( True + False\right)} $$

$$ RNP= Rate\  of\  Negative\  Prediction = \frac{\  True\kern0.5em  Negative s + False\  Negative s}{\left( True\  Prediction s + False\  Prediction s\right)} $$

$$ ACC= Accuracy\  of\  Classifier = \frac{\  True\kern0.5em  Positives + True\  Negatives}{\left( True\  Predictions + False\  Predictions\right)} $$

$$ MCC=\frac{\left( True\  Positives\ *\  True\  Negatives\right)\left( False\  Positives\ *\  False\  Negatives\right)}{\sqrt{\left( True\  Positives+ False\  Positives\right)\left( True\  Positives+ False\  Negatives\right)\left( True\  Negatives+ False\  Positives\right)\left(\  True\  Negatives+ False\  Negatives\right)}}. $$

$$ FMeasure=F\_ score= 2 \times \frac{\left( Precision* Recall\right)}{\left( Precision+ Recall\right)} $$

Several illustrative examples below demonstrate different features of PROPER. An example presented in Fig. 2 illustrates PROPERs functions, i.e. optimization, comparison, and visualization, applied to independent training and testing sets of data from a study on prediction of protein sequence crystallizability [4]. In this study, we have used a dataset of 5691 protein sequences in negative set and 4924 protein sequences in positive set. For each protein sequence 48 different features were calculated and fed into machine learning methods. This data is available at http://ffas.burnham.org/XtalPred/help.html. After loading the data, optimization of model’s structure, e.g. selection of ANN learning algorithm, is performed by generating two- and three-dimensional performance curves and then similar curves are generated to compare performance of different optimized models. ANN training begins with initial random weights for each feature and, after each iteration, a learning algorithm changes these weights to reach the highest level of accuracy. Figure 2(a-d) shows differences in performance of four standard learning algorithms applied to training of ANN on this database. More examples and detailed information about installing PROPER is available from user manual that could be downloaded from the distribution directory at sourceforge.

## Conclusions

In summary, PROPER is a freely available package for performance visualization, comparison and optimization of scoring classifiers in MATLAB. Performance visualization can be applied to output of any scoring classifier available or not available in PROPER. PROPER will be helpful in improving reproducibility and standardization of research in the field of biological Big Data outcome prediction.

## Availability and requirements

- Project name: ROPER
- https://sourceforge.net/projects/PROPER-Package
- Operating system(s): Platform independent
- Other requirements: It requires the MATLAB Statistics Toolbox
- Any restrictions to use by non-academics: None

## Additional file

Additional file 1:
**Proper user manual**. (DOCX 932 kb)

## References

[1] Liu J, Ji S, Ye J. SLEP: Sparse Learning with Efficient Projections. Arizona State University. 2009. http://www.public.asu.edu/~jye02/Software/SLEP/citation.htm.

[2] Breiman L (2001). Random Forests. Mach Learn.

[3] Hall M, Frank E, Holmes G, Pfahringer B, Reutemann P, Witten IH. The WEKA Data Mining Software: An Update; SIGKDD Explorations. 2009; Volume 11, Issue 1. http://dl.acm.org/citation.cfm?id=1656278.

[4] Jahandideh S, Jaroszewski L, Godzik A (2014). Improving the chances of successful protein structure determination with a random forest classifier. Acta Cryst D.

