# Supplementary material for: PROPER: Performance visualization for optimizing and comparing ranking classifiers in MATLAB
Source: Source Code Biol Med. 2015 Dec 3;10:15. doi: 10.1186/s13029-015-0047-1 (PMC4668683; doi:10.1186/s13029-015-0047-1)
Supplement: Additional file 1: — Proper user manual. (DOCX 932 kb) [file 13029_2015_47_MOESM1_ESM.docx]

PROPER: Performance visualization for optimizing and comparing ranking classifiers in MATLAB

Samad Jahandideh^1^, Fatemeh Sharifi^2^, Lukasz Jaroszewski^1^ and Adam Godzik^1^

^1^Bioinformatics and Systems Biology Program, Sanford-Burnham Medical Research Institute, 10901 N Torrey Pines Rd, La Jolla, California, 92307, United States

^2^Division of Computer Science, School of Informatics and Computing, Indiana University, Bloomington, IN 47405, USA

**---------------------------------------------------------------------**

**Version:** 1.0

**Date:** 2015-03-17

**License:** GPL

**URL:** http://sourceforge.net/projects/proper-package

**Requirement:** MATLAB Statistics Toolbox

**Maintainer:** Samad Jahandideh <sjahandideh@burnham.org or samad_jahandideh@yahoo.com>

**Platforms:** MS Windows, Mac OS, Unix/Linux

***1. Prediction* function**

The *prediction* function which is located in scoring-methods folder performs training and testing of existing classifiers in this package through three different evaluation methods. The proposed optimal workflow is to start with a single classifier, optimize the structure of that classifier, and then implement the other classifiers. After the training process, you can easily save the output and optimized model.

**1.1. Evaluation procedure**

Three different standard evaluation methods, namely re-substitution, independent validation, and cross-validation, are implemented in this package. Although application of re-substitution method (i.e. training and testing of classifier on the same database) results in highly biased prediction, this method can examine the relevance of input features to the problem of interest. Two separate training and testing sets are needed for the independent method.

*Getting Started:*

*Step 1: Data selection and import*

First, set the *Prediction/Scoring-methods* directory as the MATLAB current folder and transfer the data to *Data* folder. Data could be a single file or two separated training and testing data files. The dataset should be in a simple N*M matrix format, in which rows are samples and columns are features, with exception of the last column which contains the labels of each sample (see sample datasets).

*Step 2: Evaluation method selection*

Next, either run *Prediction*.m or type *Prediction* in the Command Window. After a short query about the model of interest and selected dataset, an evaluation method will be requested. If one dataset will be used such that the program will split the data into training and testing sets, enter either ‘CV’ for cross-validation or ‘RESUB’ for re-substitution. Otherwise, enter ‘IND’ for independent validation of two separate datasets defined as a training set and test set, respectively.

*Step 3: Training set selection and naming conventions*

During the next step, the program requests entry of the Training set name. If the name of dataset is “Trainingset” then add the .mat suffix and enter *Trainingset.mat*. Next, the program requests the name of the Testing set (hence, enter *Testingset.mat* for dataset “Testingset”). For a Cross Validation procedure, only the name of one dataset is needed; enter the name of the dataset with the .mat suffix. For example, if the data is named as “Sample”, enter *Sample.mat*. The program splits the data into training and testing sets.

*Step 4:* Evaluation *selection and execution*

If CV is selected for the evaluation method, two options will be available: i) K-fold cross validation and ii) leave-one-out cross validation. K-fold cross-validation divides the data into K sets, of which the program uses a combination of K-1 sets to train the model, and uses the remainder set for testing purpose. Repeating the process K times, the program uses a different combination of K-1 sets as its training data (and therefore a different set as its testing data). To select K-fold cross validation enter ‘K’, and in the next step enter the number of folds. For example, enter ‘5’ if the data will be divided into five parts, or enter ‘10’ to divide the data into ten parts.

In the alternative leave-one-out option, each iteration of the algorithm selects one and only one datum for testing purpose and uses the other N-1 data to train the model. The program repeats this procedure N times. To use this type of partitioning enter ‘LOO’ which stands for leave-one-out.

**1.2. Classifiers**

After selecting the evaluation method, additional inputs are requested including the classifier name and some other parameters. When the program requests the name of the classifier, enter one of the following classifier abbreviations: ‘ANN’ for Artificial Neural Network (Bishop, 1995), ‘RF’ for Random Forest (Breiman, 2001) classification, or ‘RLR’ for Regularized Logistic Regression (Liu et al., 2009). After selection of classifier, depending on the selected classifier, you are required to input different parameters (Figure 1).

There are several parameters for the ANN settings including number of hidden nodes, number of iterations, training algorithm(s), and learning rate (see ANN MATLAB toolbox available at http://www.mathworks.com/products/neural-network/?refresh=true). One can use default parameters, or enter new sets of parameters. The number of training and testing procedures is equal to the product of the size of selected parameters. For example, if an ANN will be trained with three different sets of hidden nodes, two different training algorithms, and three different iterations, for an independent or re-substitution validation, the classifier will be trained and tested 3*2*3 = 18 times. For a cross validation, this number also depends on the number of folds; therefore, the model will be trained and tested *Ntm* times when *Ntm* is the product of size of parameters and validation folds. For example, with the same set of parameters in a 5-fold cross-validation, a total 18*5=90 training and testing procedures. The best way to optimize ANN structure is to find the optimum parameters one-by-one.


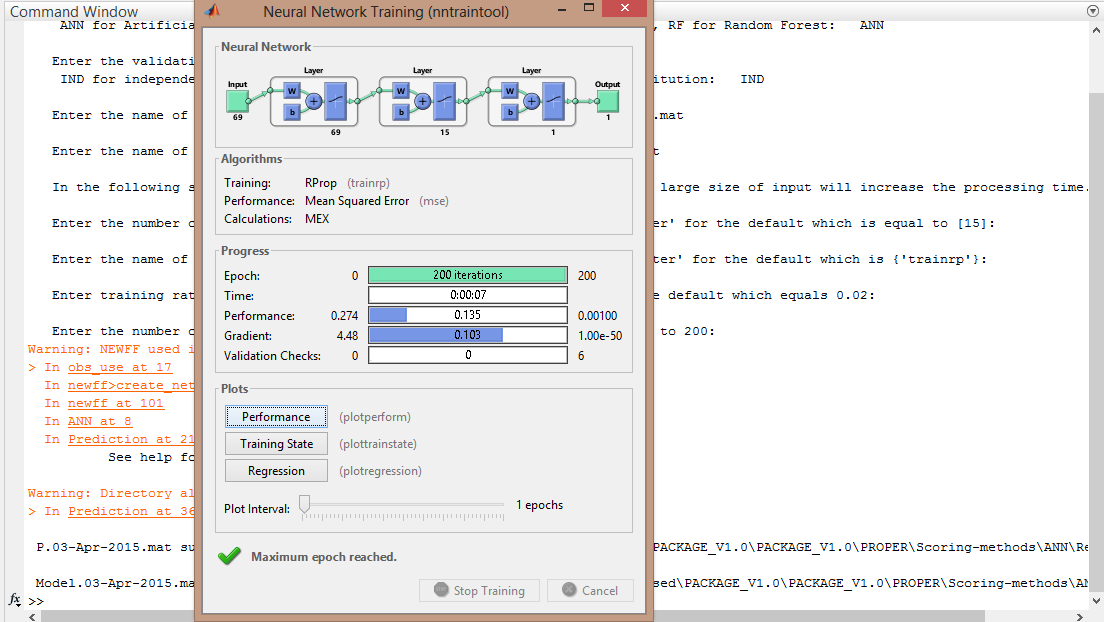


Figure 1: Training an ANN in PROPER package.

For Regularized Logistic Regression and Random Forest, in this package there is only one important parameter for each classifier, i.e. Rho for RLR which is shrinkage rate and number of trees for RF.

Figure 2 illustrates how the script requests the name of the classifier, evaluation method, database(s) and classifier parameters.


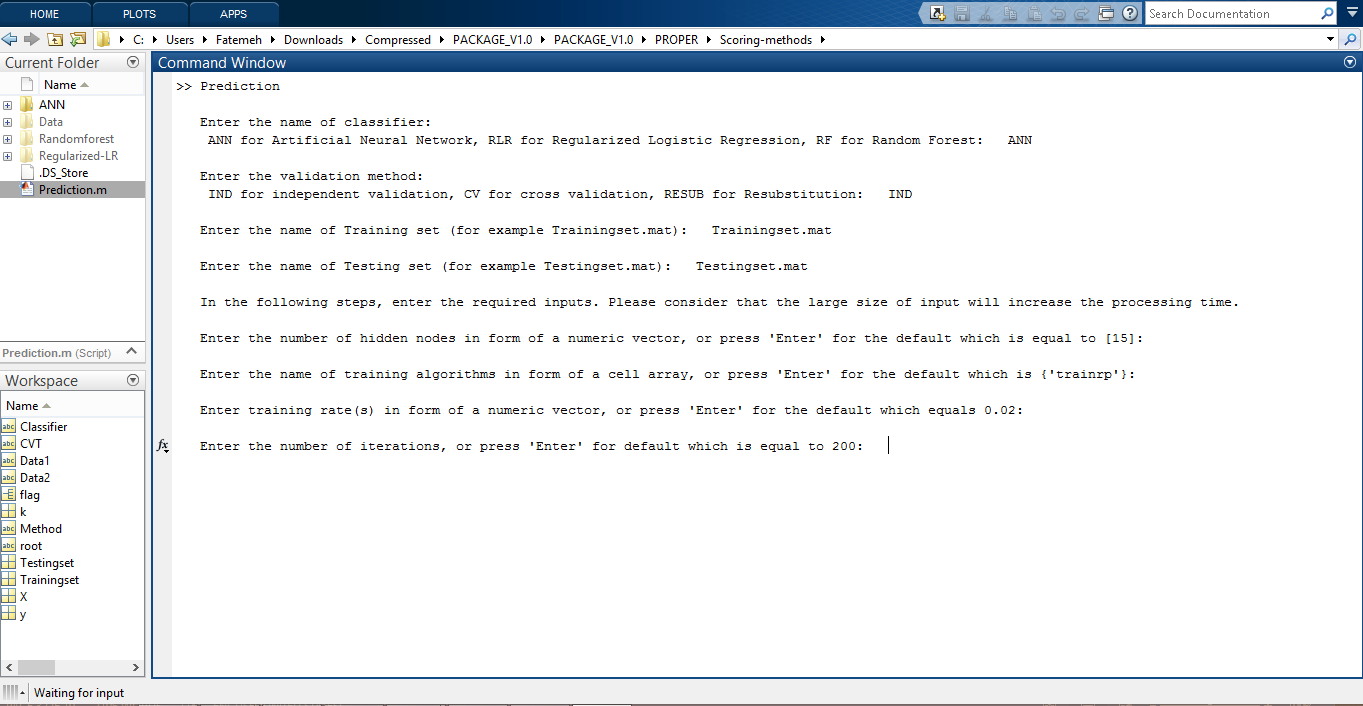


Figure 2: Prediction function requests the name of the classifier, evaluation method, database(s) and classifier parameters.

***2. Performance* function**

The simplest way to use *Performance* function is to set the *Performance* directory as the current MATLAB folder. The *Performance* function uses the output of classifiers/models to calculate 13 different performance measures across a wide spectrum of cutoffs. For this purpose, the output of classifiers/models is presented in matrix form, in which each column includes a vector of predicted values for all tested samples.

*Performance* function consists of three main sections: Normalization, Cutoffs and Indices Calculation, and Calculation of Performance Measures (Figure 3).

Figure 3: Three main sections of performance function.

**2.1. Normalization**

Variables:

*Input variables:*

- P is a matrix of values predicted by classifiers. Each column of P shows output of one specific classifier.
- n, the number of rows in matrix P, shows the number of test samples.

*Output variables:*

- Ntm, the number of columns in Matrix P, shows the Number of trained models.
- NormP stores the Normalized values of Matrix P.

Figure 4 presents a sample input which is a part of predicted values by three different classifiers on all 4885 testing samples from our sample data available in this package.


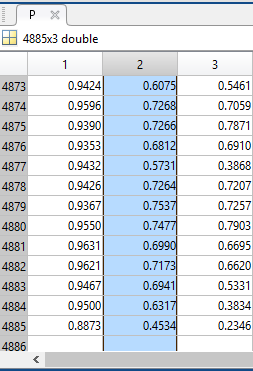


Figure 4: A sample input, P matrix, of performance function.

By using formula 1, each column of P is normalized from a domain of [Min_P_, Max_P_] into a range of [0, 1].

$NormX\left( i \right)=\frac{X(i)-Min(X)}{Max\left( X \right)-Min(X)}$

Formula 1: Normalizing Vector X

According to formula 1, if P= [3, 6, 5] then NormP= [0, 1, 0.6667].

**2.2. Cutoffs and Indices Calculation**

In binary classification, choosing a proper cutoff is of great importance; a bad cutoff can lead to a wrong prediction. Rather than choosing an arbitrary cutoff that can cause missed detection or false alarms, a great number of cutoff values are evaluated in order to help find a reasonable cutoff based on available data.

For this reason, 100 cutoff values between zero and one (*i.e.* steps of 0.01) are assigned one by one as a potential cutoff, for which four indices including True Positive, True Negative, False Positive, and False Negative are calculated. True Positives are those predictions that correctly assign a sample to its real class, while False Negatives are those ones that exclude a sample from its real class. False Positives are those predictions that assign a sample to a wrong class, while True Negatives are those ones that correctly exclude a sample from wrong classes.

**2.3. Calculation of Performance Measures**

We calculate 13 different performance measures that are used to comprehensively evaluate the performance of classifiers/models:

$\boldsymbol{T=True Predictions=(True Positive+True Negative)}$

$\boldsymbol{F=False Predictions=(False Positive+FalseNegative)}$

$$\boldsymbol{TPR=Sensitivity=Recall=}\frac{\boldsymbol{True Positive}}{\boldsymbol{(True Positive + False Negative)}}$$

$$\boldsymbol{FNR=False Negative Rate=}\frac{\boldsymbol{False Negative}}{\boldsymbol{(True Positive + False Negative)}}$$

$$\boldsymbol{FPR=False Positive Rate=Fallout=}\frac{\boldsymbol{False Positive}}{\boldsymbol{(True Negative + False Positive)}}$$

$$\boldsymbol{TNR=Negative Negative Rate=Specificity=}\frac{\boldsymbol{True Negative}}{\boldsymbol{(True Negative + False Positive)}}$$

$$\boldsymbol{PPV=Positive Predictive Value=}\frac{\boldsymbol{True Positive}}{\boldsymbol{(True Positive + False Psitive)}}$$

$$\boldsymbol{NPV=Negative Predictive Value=}\frac{\boldsymbol{True Negative}}{\boldsymbol{(True Negative + False Negative)}}$$

$$\boldsymbol{RPP=Rate of Positive Prediction=}\frac{\boldsymbol{True Positive + False Positive}}{\boldsymbol{(True + False)}}$$

$$\boldsymbol{RNP=Rate of Negative Prediction=}\frac{\boldsymbol{True Negative+ False Negative}}{\boldsymbol{(True + False)}}$$

$$\boldsymbol{ACC=Accuracy of Classifier=}\frac{\boldsymbol{True Posi}\boldsymbol{tive + True Negative}}{\boldsymbol{(True +False )}}$$

$$\boldsymbol{MCC=}\frac{\left( \boldsymbol{True Positive * True Negative} \right)\left( \boldsymbol{False Positive * False Negative} \right)}{\sqrt{\left( \boldsymbol{True Positive+False Positive} \right)\left( \boldsymbol{True Positive+False Negative} \right)\left( \boldsymbol{True Negative+False Positive} \right)\left( \boldsymbol{True Negative+False Negative} \right)}}\boldsymbol{.}$$

$$\boldsymbol{FMeasure=F\_score=2\times}\frac{\boldsymbol{(Precision*Recall)}}{\boldsymbol{(Precision+Re}\boldsymbol{call)}}$$

**Usage**

***Performance(p,labels)***

**Example**

Drag P, the predicted values for all tested samples, and Labels Matrices that are located in Performance directory into Workspace and type ‘*Performance(p, labels)’* in MATLAB Command Window. This function will create a new folder named results in the Performance directory, and save a structure named M that consists of all thirteen calculated performance measures per each single cutoff. The date of creation of results will be saved as part of structure’s name. By dragging the structure into MATLAB Workspace, and double clicking on it you will be able to see *T, F, TPR*, and other measure values.

M=Performance(P,Labels)

M =
 T: [100x3 double]
 F: [100x3 double]
 TPR: [100x3 double]
 FNR: [100x3 double]
 FPR: [100x3 double]
 TNR: [100x3 double]
 PPV: [100x3 double]
 NPV: [100x3 double]
 RPP: [100x3 double]
 RNP: [100x3 double]
 ACC: [100x3 double]
 MCC: [100x3 double]
 FM: [100x3 double]

**3. Visualization**

To enable evaluation of the performance for a specific model, or a group of models/classifiers, two user-friendly MATLAB functions named Visualization2D and visualization3D are provided to generate two- and three-dimensional performance curves based on calculated performance measures. Some of these indices can individually evaluate the performance of the classifier. For example, the more the MCC value is the more qualified the classifier, or the higher the amount of accuracy is the more accurate the classifier. These kinds of indices can be plotted against their corresponding cutoff in order to help find a preferred cutoff, and obtain the maximum performance of a model or models.

The visualization function provides options for plotting any possible combination of two of these indices. Analyzing an adequate number of these curves enables finding an optimum cutoff that maximizes the performance of one or more classifiers. Two- and three-dimensional plotting options are provided in this package to allow comparison of multiple performance measures. A straightforward function is available to plot a variety of 3D plots based on your choice of measures. It is possible to plot 1000 different plots by using the Visualization3D function, for instance, MCC vs. ACC vs. CUTOFF, SENSITIVITY vs. PRECISION vs. SPECIFICITY, etc.

**3.1. Visualization2D**

**Usage**

***Visualization2D(M, DOUBLE)***

**Example**

Drag M Matrix, which is located in the Performance/results directory, into Workspace, and change the path to the *visualization* directory. M includes all performance measures’ values throughout all cutoffs. Then double click on the DOUBLE variable and define a combination of measures for each single 2D plot (see Figure 5). The first column shows the performance measure which will appear on X axis, and second column shows the performance measures that will appear on Y axis. Each row is corresponding to one 2D plot.

***
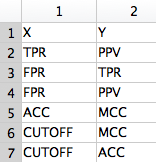
***

Figure 5: DOUBLE variable example.

Running Visualization2D function on combinations of measures presented in the previous table for evaluating three different models produced the following plots:

Figure 6: The output of Visualization2D function.

**3.2. Visualization3D**

**Usage**

***Visualization3D(M, TRIPLE)***

**Example**

Similar procedures as mentioned in the previous section can be followed to draw 3D plots. Double click on TRIPLE variable in MATLAB workspace, and define a combination of measures for each single 3D plot (see Figure 7). The first column shows the performance measures that will appear on X axis, the second column shows the performance measures that will appear on Y axis, and the third column shows the performance measures that will appear on Z axis. Each row corresponds to one 3D plot.


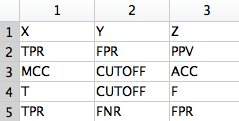


Figure 7: TRIPLE variable example.

If the Visualization3D function is run on combinations of measures presented in the previous table for three different models, the following plots are output:

Figure 8: The output of Visualization3D function.

**References**

Liu, J., Ji S. and Ye J. (2009) SLEP: Sparse Learning with Efficient Projections. Arizona State University.

Breiman, L. (2001) Random Forests. Machine Learning, 45:5-32.

Bishop, C. M. (1995). Neural Networks for Pattern Recognition. Oxford: Clarendon Press.
